# Targeting the JAK/STAT pathway in atopic dermatitis

**DOI:** 10.3389/fimmu.2026.1757562

**Published:** 2026-03-26

**Authors:** Lixia Cui, Pengyue Liu, Kun Wu, Xiuping Han, Ge Peng

**Affiliations:** 1 Department of Dermatology and Venereology, The Second Affiliated Hospital of Hainan Medical University, Haikou, Hainan, China; 2 Department of Dermatology, Shengjing Hospital of China Medical University, Shenyang, Liaoning, China; 3 Atopy (Allergy) Research Center, Juntendo University Graduate School of Medicine, Tokyo, Japan

**Keywords:** atopic dermatitis, biologic therapy, immunopathogenesis, JAK inhibitors, JAK/STAT signaling, skin inflammation

## Abstract

Atopic dermatitis (AD) is a chronic, inflammatory skin disorder characterized by immune dysregulation, skin barrier dysfunction, and pruritus. Central to its pathogenesis is the Janus kinase/signal transducer and activator of transcription (JAK/STAT) signaling pathway, which mediates cytokine responses—including interleukin (IL)-4, IL-13, IL-31, and thymic stromal lymphopoietin—that drive T-helper 2-skewed inflammation and epidermal barrier impairment. In recent years, the therapeutic landscape of AD has been transformed by the development of JAK inhibitors, offering both systemic and topical treatment options for patients unresponsive to conventional therapies. This narrative review provides a comprehensive overview of the JAK/STAT pathway’s biological role in AD, including its regulation of immune responses and skin inflammation. We summarize current JAK inhibition therapies under clinical use or investigation, compare their efficacy and safety profiles, and examine unresolved controversies surrounding long-term outcomes and adverse effects. Furthermore, we explore future innovations in JAK/STAT research, including precision medicine approaches, third-generation allosteric inhibitors, microbiome-informed strategies, and advanced drug delivery technologies. Collectively, understanding and refining JAK/STAT-targeted therapy hold great promise for individualized, safe, and effective management of AD.

## 1 Introduction

Atopic dermatitis (AD) is a chronic, relapsing, pruritic, and inflammatory skin disease that commonly begins in early childhood and often persists into adulthood. It affects up to 20% of children and 10% of adults worldwide, posing a significant burden on quality of life and healthcare systems (1, 2). Clinically, AD is characterized by eczematous lesions, skin dryness, lichenification, and intense itching. Histopathologically, AD presents with epidermal hyperplasia, immune cell infiltration, and altered skin architecture.

The pathogenesis of AD is complex and multifactorial, involving genetic predisposition, environmental exposures, epidermal barrier dysfunction, and immune dysregulation (3, 4). Among these, the breakdown of the skin barrier—exemplified by filaggrin mutations and increased transepidermal water loss—facilitates allergen penetration and microbial colonization (5). Concurrently, immune imbalance plays a pivotal role in AD pathogenesis, characterized by type-2–skewed inflammation involving both adaptive and innate immune responses. While T-helper 2 (Th2) cells contribute substantially to the production of cytokines such as interleukin (IL)-4, IL-13, and IL-31, group 2 innate lymphoid cells (ILC2) also play a key role in amplifying type-2 inflammatory signaling and promoting allergic inflammation. Together, these cellular components drive pruritus, inflammation, and epidermal barrier dysfunction in AD (6–8). Chronic lesions additionally involve Th1, Th17, and Th22 responses. Moreover, dysbiosis of the skin microbiome, particularly the overgrowth of *Staphylococcus aureus*, exacerbates inflammation and disrupts immune homeostasis (9). Beyond cutaneous manifestations, AD is frequently associated with allergic comorbidities and is widely regarded as the first step of the “atopic march”, a progression in which AD may precede food allergy, allergic rhinitis, and asthma. Early epidermal barrier dysfunction and type-2 inflammatory responses are thought to promote systemic allergic sensitization, linking AD to these subsequent atopic conditions. This broader disease context highlights that AD represents not only a skin disorder but also part of a systemic allergic spectrum with important clinical implications (10).

At the core of this immunopathology lies the Janus kinase (JAK)/signal transducer and activator of transcription (STAT) pathway, a highly conserved signaling cascade essential for transducing extracellular cytokine signals into gene expression changes. This pathway is engaged by numerous AD-related cytokines, including IL-4, IL-13, IL-31, and thymic stromal lymphopoietin (TSLP), making it a central node in disease propagation (11, 12).

Advances in our understanding of JAK/STAT signaling have revolutionized therapeutic approaches in multiple immune-mediated disorders. In AD, the development of selective JAK inhibitors (e.g., abrocitinib, upadacitinib, ruxolitinib) has provided promising alternatives to conventional immunosuppressants and biologics (13). JAK1-selective inhibition, in particular, targets key pathways involved in Th2-mediated inflammation and pruritus signaling, while potentially reducing off-target effects associated with pan-JAK inhibitors (14, 15).

This narrative review aims to synthesize current mechanistic and clinical evidence regarding the JAK/STAT pathway in AD. Relevant literature was identified through searches of major biomedical databases, including PubMed, Web of Science, and Google Scholar, using combinations of keywords such as “atopic dermatitis”, “JAK/STAT pathway”, “Janus kinase”, “JAK inhibitors” and “cytokine signaling”. Priority was given to recent studies, clinical trials, and influential mechanistic investigations related to JAK/STAT signaling and its therapeutic targeting in AD. We begin by outlining the fundamental biology of JAK/STAT signaling, then explore its specific roles in AD pathogenesis, highlight current and emerging therapeutic strategies, address unresolved controversies, and propose future directions for translational research and precision medicine in this field.

## 2 Biology of JAK/STAT signaling

### 2.1 Overview of the JAK/STAT signaling pathway

The JAK/STAT pathway is a highly conserved and rapid signaling mechanism that transmits extracellular cytokine signals to the nucleus, resulting in transcriptional regulation of genes involved in immunity, cell proliferation, differentiation, and apoptosis. This pathway consists of four JAK family members (JAK1, JAK2, JAK3, and tyrosine kinase 2 (TYK2)) and seven STAT transcription factors (STAT1-4, STAT5a, STAT5b, and STAT6), each with distinct but often overlapping functions in cellular signaling networks (16).

Signal initiation begins with the binding of specific cytokines, interferons, or growth factors to their cognate receptors on the cell surface. This interaction induces conformational changes that bring JAKs into proximity, leading to their activation through trans-phosphorylation. Activated JAKs subsequently phosphorylate tyrosine residues on the cytoplasmic tails of the receptors, which serve as docking sites for STAT proteins. Upon recruitment, STATs are phosphorylated by JAKs, undergo dimerization, and translocate into the nucleus, where they bind to specific DNA sequences to regulate gene expression (17). As transcription factors, STAT proteins control the expression of numerous immune-related genes, including cytokines, chemokines, and regulators of cell survival and proliferation.

In the context of AD, this pathway plays a critical role in mediating immune signals. JAK1, in particular, is activated by key Th2 cytokines such as IL-4, IL-13, and IL-31, which are central to AD pathogenesis. These cytokines signal predominantly through the JAK1/STAT6 axis, leading to the upregulation of genes associated with inflammation, immunoglobulin (Ig) E production, and skin barrier disruption (18, 19). Notably, IL-31, a pruritogenic cytokine, activates both JAK1 and JAK2, culminating in STAT3 and STAT5 phosphorylation and contributing to the intense itch observed in AD patients (11, 20). Thus, the functional specificity of different JAK and STAT proteins forms the molecular basis for therapeutic targeting in AD.

### 2.2 JAK/STAT signaling in immune system regulation

The JAK/STAT pathway serves as a central node in immune system regulation, orchestrating both innate and adaptive responses. In innate immunity, JAK/STAT signaling is essential for the action of type I and II interferons, which regulate the activity of macrophages, dendritic cells, and natural killer cells during pathogen recognition and elimination (21). For example, interferon-gamma (IFN-γ) activates JAK1 and JAK2, leading to STAT1 phosphorylation and subsequent transcription of genes related to antigen presentation and microbial clearance (22). In adaptive immunity, the JAK/STAT pathway is critical for the differentiation of CD4+ Th cell subsets. STAT6 mediates Th2 polarization in response to IL-4 and IL-13, driving the hallmark allergic phenotype of AD (23–25). Through this process, the JAK/STAT pathway also contributes to cytokine production, as differentiated Th2 cells produce IL-4, IL-5, and IL-13, thereby reinforcing type 2 inflammatory responses. STAT3 is involved in the differentiation of Th17 cells, which are more prominent in chronic AD lesions or in patients with intrinsic or Asian AD subtypes, and promotes the production of cytokines such as IL-17 and IL-22 (25–27). Additionally, STAT5a/b is activated by IL-2 signaling and supports the survival of regulatory T cells, which are often dysfunctional in AD patients (28). These immune mechanisms are tightly regulated, and dysregulation of the JAK/STAT axis can lead to sustained inflammation, epidermal hyperplasia, and impaired barrier repair in AD. Transcriptomic studies have revealed elevated expression of phosphorylated STAT6 and STAT3 in AD skin, further implicating this pathway in disease progression (29). Thus, JAK/STAT signaling is not merely a downstream effector but an active amplifier of the inflammatory cascade in AD.

### 2.3 Genetic regulation of the JAK/STAT pathway

Genetic variants in the JAK/STAT signaling components or their upstream regulators can significantly influence susceptibility to immune-mediated diseases, including AD. For instance, genome-wide association studies have identified polymorphisms in genes encoding IL4RA, IL13, and STAT6 that are associated with increased risk for allergic diseases such as asthma, eczema, and AD (30–32). These variants may lead to enhanced signaling through the JAK1/STAT6 axis, resulting in exaggerated Th2 immune responses and increased IgE synthesis (32, 33). Moreover, copy number variations and non-coding single nucleotide polymorphisms in regulatory elements of the JAK/STAT pathway have been implicated in modulating skin inflammation and responsiveness to cytokine signaling. In particular, polymorphisms in TYK2 have been linked to altered interferon and interleukin signaling in several autoimmune conditions, highlighting the broader immunogenetic landscape of JAK/STAT dysregulation (34, 35). While somatic mutations such as JAK2 V617F are well-documented in myeloproliferative neoplasms (36), such mutations are not commonly reported in AD. Nevertheless, subtle germline polymorphisms or epigenetic modifications affecting JAK/STAT components may contribute to interindividual variability in disease severity and treatment response. These findings underscore the need for integrating genomic data with clinical phenotyping to develop personalized therapeutic approaches targeting the JAK/STAT pathway in AD.

## 3 JAK/STAT pathway in AD

### 3.1 Role of the JAK/STAT pathway in AD pathogenesis

The JAK/STAT signaling pathway plays a central and multifaceted role in the pathogenesis of AD, influencing immune dysregulation, epidermal barrier dysfunction, and neuroimmune interactions. In particular, Th2-type cytokines such as IL-4, IL-13, and IL-31—hallmarks of AD inflammation—signal through JAK1- and JAK3-mediated phosphorylation of STAT6 and STAT3, promoting the transcription of genes that drive allergic inflammation, tissue remodeling, and pruritus (37–39). In keratinocytes, IL-4 and IL-13 signaling via the JAK1/STAT6 axis downregulates the expression of key barrier proteins such as filaggrin and loricrin, contributing to skin barrier dysfunction and increased susceptibility to allergens and pathogens (5, 40). This inhibition occurs through STAT6 competition with activator protein 1 for the transcriptional coactivator p300/CBP, thereby repressing epidermal differentiation programs (41). Furthermore, IL-31, which signals through JAK1 and JAK2, directly promotes itch by activating neuronal pathways and enhancing the expression of pruritogenic mediators in the skin (42).

Moreover, recent immunohistochemical and transcriptomic studies have provided compelling evidence of JAK/STAT pathway activation in human AD lesions. Robust upregulation of genes downstream of the JAK/STAT pathway, including *STAT1*, *STAT3*, and *SOCS3*, correlated with clinical severity scores such as Scoring of AD (SCORAD) and Eczema Area and Severity Index (EASI), suggesting that JAK/STAT pathway activation reflects disease burden of AD (43, 44). Complementary immunohistochemical staining of lesional skin confirmed increased phosphorylation of STAT1 and STAT3 in both epidermal keratinocytes and dermal immune infiltrates, supporting functional activation of the pathway *in situ* (39). Importantly, the extent and nature of JAK/STAT activation appear to differ between acute and chronic phases of AD. Acute lesions are dominated by Th2-type inflammation, with strong upregulation of IL-4 and IL-13 signaling via the JAK1/STAT6 axis. This leads to suppression of epidermal differentiation markers and intensification of barrier defects. In contrast, chronic AD lesions exhibit a more complex cytokine environment, including contributions from Th1 (via IFN-γ and STAT1), Th17 (via IL-22 and STAT3), and Th22 pathways (45). Immunohistochemical studies reveal higher levels of pSTAT3 in chronic AD, reflecting sustained activation of epidermal proliferation and remodeling programs (46). These differences underscore the dynamic nature of JAK/STAT signaling across AD stages and support the rationale for tailoring JAK inhibitor treatment strategies based on disease chronicity and molecular phenotype.

**Figure 1:**
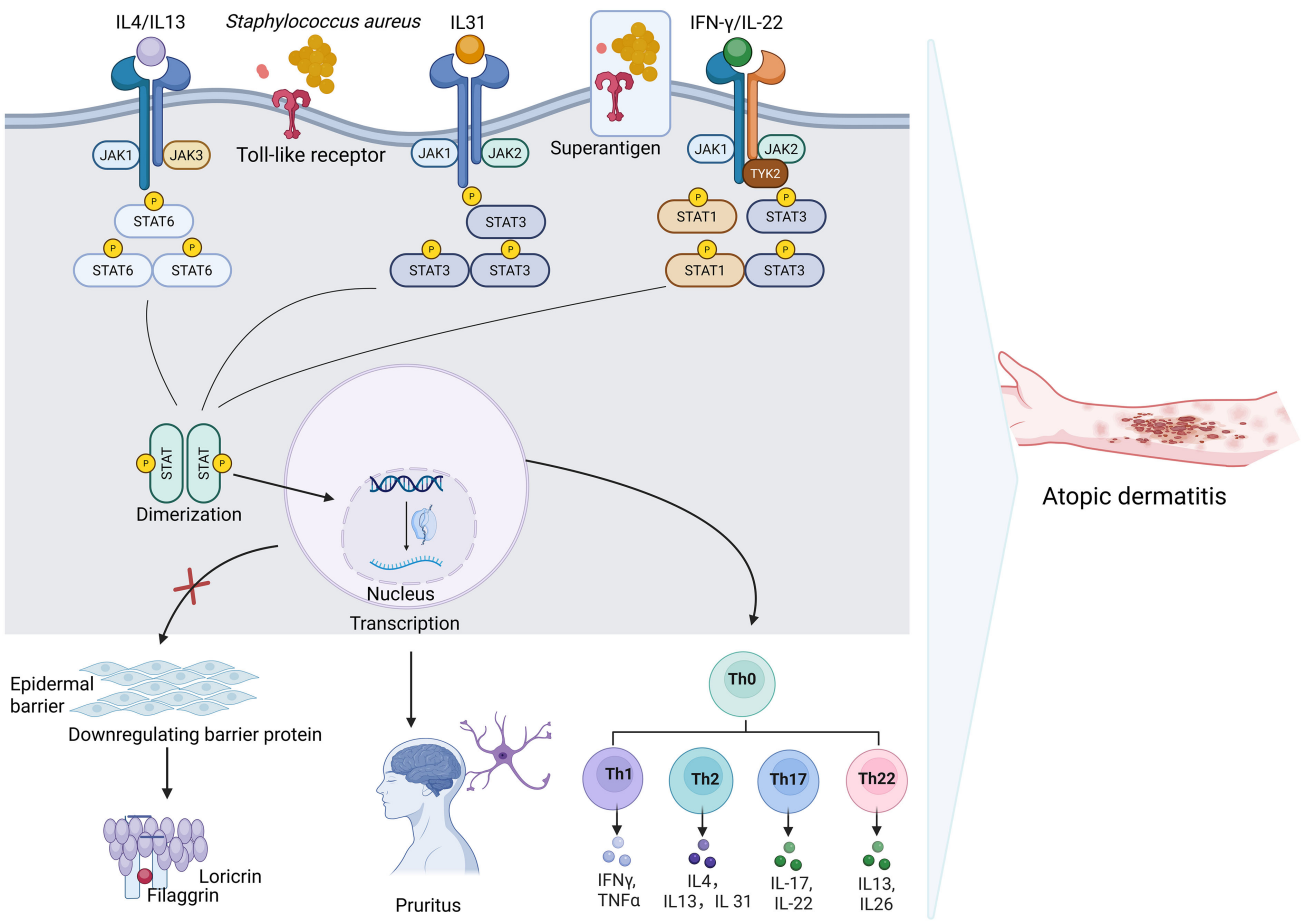
The JAK/STAT signaling pathway in the pathogenesis of AD. Environmental triggers and *Staphylococcus aureus* act on a disrupted skin barrier, leading to the release of multiple cytokines (IL-4, IL-13, IL-31, IFN-γ, IL-22), which activate JAKs (JAK1, JAK2) and downstream STATs (STAT6, STAT3, STAT1). Activated STATs dimerize and translocate to the nucleus to regulate gene transcription, resulting in key pathological outcomes including Th2/Th1/Th17/Th22-driven inflammation, downregulation of barrier proteins such as filaggrin, loricrin, as well as induction of pruritus.

Additionally, recent research has also linked the JAK/STAT pathway to skin microbiome interactions in AD. Over-colonization by *S.aureus*, a common feature in AD lesions, can further amplify inflammation through superantigen production and Toll-like receptor engagement, leading to cytokine release that feeds into JAK-mediated signaling cascades (47). In this context, JAK/STAT activation integrates environmental, microbial, and immune triggers, exacerbating the chronic inflammation typical of AD (**Figure 1**).

### 3.2 Biomarkers related to the JAK/STAT pathway in AD

Several molecular and cellular biomarkers associated with the JAK/STAT pathway have been identified in AD, offering potential insights into disease activity, severity, and treatment response. Among them, elevated serum and lesional levels of IL-4, IL-13, IL-31, and TSLP—all of which rely on JAK-dependent signaling—are considered reliable markers of Th2-dominated inflammation and are directly linked to the severity of pruritus and eczema (48, 49). Meanwhile, transcriptomic profiling of lesional AD skin has shown upregulation of phosphorylated STAT3 and STAT6, correlating with disease severity and inflammatory gene expression signatures (50). In addition, periostin, a matricellular protein downstream of IL-13 signaling, has emerged as a key pruritogenic biomarker regulated via the JAK/STAT pathway in keratinocytes. Periostin not only promotes itch but also drives fibrosis and epithelial-mesenchymal transition, contributing to lichenification and skin remodeling in chronic AD (51).

Animal models have also demonstrated JAK/STAT-dependent signatures in response to immunomodulatory therapy. For instance, treatment of AD-like mice with human umbilical cord-derived mesenchymal stem cells suppressed JAK-mediated IL-4 and IL-17 receptor expression, supporting the value of downstream gene expression profiles as dynamic biomarkers of therapeutic efficacy (52). Genetic biomarkers also play a role. Gain-of-function variants in STAT3, identified in rare immune dysregulation syndromes, are associated with altered chemokine expression such as CXCL8, suggesting potential molecular patterns of exaggerated STAT activation (53–55).

## 4 Therapeutic strategies targeting the JAK/STAT pathway

### 4.1 Current therapies modulating the JAK/STAT pathway in AD

Targeting the JAK/STAT signaling cascade has emerged as a promising therapeutic strategy in AD, particularly due to the central role of this pathway in mediating Th2-driven inflammation and pruritus. JAK inhibitors are small-molecule compounds that block the enzymatic activity of JAK, thereby preventing downstream STAT phosphorylation and nuclear translocation. This leads to broad suppression of cytokine signaling, including IL-4, IL-13, IL-31, and TSLP, which are central mediators in the pathophysiology of AD (56). There are both topical and systemic JAK inhibitors, each offering distinct advantages depending on disease severity, treatment goals, and patient profile.

In the context of systemic JAK inhibitors, tofacitinib, a pan-JAK inhibitor with relative selectivity for JAK1 and JAK3, has shown efficacy in small case series involving refractory eczematous dermatoses, including AD. In a cohort of AD patients unresponsive to topical steroids or calcineurin inhibitors, tofacitinib administration led to significant improvement in disease severity scores within four weeks (57). However, its long-term use is limited by safety concerns such as infection risk and laboratory abnormalities. Furthermore, abrocitinib and upadacitinib, both selective JAK1 inhibitors, have been approved for moderate-to-severe AD in adults and adolescents. In the phase III trials such as JADE MONO-1 and MONO-2, abrocitinib showed superior efficacy to placebo and comparable performance to dupilumab, with rapid onset of action (58, 59). The oral formulation of abrocitinib allows for systemic immune modulation, making it suitable for widespread or recalcitrant disease, although dose-dependent adverse events, including nausea and acne, have been reported (60, 61). Upadacitinib, another selective JAK1 inhibitor, has similarly shown high efficacy in phase III trials (MEASURE UP 1/2). Importantly, these trials were conducted during the COVID-19 pandemic, which may have influenced certain aspects of study conduct and patient management, although the overall efficacy outcomes remained consistent with expectations for JAK1 inhibition (62). In network meta-analyses, high-dose upadacitinib (30 mg/day) ranked among the most effective agents in achieving skin clearance and reducing itch severity, outperforming both dupilumab and baricitinib (63). However, its potent immunosuppressive effects raise concerns regarding infections, thrombosis, and laboratory abnormalities, highlighting the need for careful patient selection and monitoring (63–66). Ivarmacitinib, a newly developed selective JAK1 inhibitor, has demonstrated significant clinical efficacy in a phase III randomized clinical trial for AD and has been approved in China for the treatment of the patients with moderate-to-severe AD. A topical formulation of ivarmacitinib is also undergoing regulatory review for AD, offering an additional therapeutic modality with the potential for reduced systemic exposure (67, 68). Finally, baricitinib, a JAK1/JAK2 inhibitor, has also been shown to reduce AD severity with acceptable tolerability, although its use in AD is limited to select countries (69–71).

Topical JAK inhibitors are also under development to address the limitations of systemic therapy. Ruxolitinib, a JAK1/JAK2 inhibitor, has shown efficacy in mild-to-moderate AD. Its cream formulation (1.5%) is approved in the United States for short-term and non-continuous chronic treatment. Pharmacokinetic studies have demonstrated minimal systemic absorption, with plasma concentrations remaining below the thresholds associated with systemic JAK inhibition and its adverse effects (72–75). This safety profile makes ruxolitinib cream particularly attractive for patients concerned about systemic immunosuppression. Moreover, delgocitinib (JTE-052), a pan-JAK inhibitor ointment approved in Japan, has shown promising efficacy and safety in both pediatric and adult AD populations in Japan. In phase I/II studies, delgocitinib significantly improved EASI and pruritus scores without detectable systemic exposure, making it an attractive option for long-term maintenance therapy (76–80). Brepocitinib, a TYK2/JAK1 inhibitor, is currently under investigation in topical formulation. In a phase II trial, brepocitinib demonstrated efficacy and tolerability in patients with mild-to-moderate AD. Notably, once-daily dosing achieved similar outcomes to twice-daily administration, suggesting a convenient therapeutic regimen (81). In addition, cerdulatinib and gusacitinib, both dual spleen tyrosine kinase (SYK)/JAK inhibitors, are being studied for their therapeutic potential in AD. These agents represent a new class of dual-pathway inhibitors that could offer broader immunomodulatory effects by targeting both JAK and SYK signaling, which may further modulate inflammatory pathways relevant to AD pathogenesis (82–84).

In addition to synthetic inhibitors, phytochemicals such as baicalin—a flavonoid derived from *Scutellaria baicalensis*—have demonstrated JAK/STAT-modulating effects in preclinical AD models. Baicalin was shown to reduce inflammation and improve skin barrier function in mice through downregulation of STAT3 and STAT6 phosphorylation, modulation of gut-skin axis via microbiome restoration, and reduction of IL-4/IL-13 expression (18). These findings suggest the potential of plant-derived compounds as complementary or adjunctive therapies targeting JAK/STAT signaling.

Although not directly classified as JAK inhibitors, biologics such as dupilumab (anti-IL-4Rα) and tralokinumab (anti-IL-13) indirectly modulate JAK/STAT signaling by blocking the cytokines that activate this pathway. These agents remain effective and well-tolerated in many patients, and combination or sequential therapy strategies with JAK inhibitors are being explored (11, 85).

### 4.2 Comparative analysis of JAK inhibitors in AD management

A key advantage of JAK inhibitors lies in their broad-spectrum cytokine suppression compared to the target-specific action of biologics. However, JAK inhibitors differ in their kinase selectivity, route of administration, and risk profiles, which influence clinical decision-making. **Table 1** summarizes key features of commonly used JAK inhibitors in AD. In addition, **Table 2** summarizes practical considerations that may assist clinicians in selecting JAK inhibitors or alternative targeted therapies based on individual patient characteristics and clinical scenarios.

**Table 1:** Key features of JAK inhibitors in AD clinical trails.

Drug	Target	Phase	Onset	EASI-75 response	Notable AEs	Refs
Oral JAK inhibitors						
Abrocitinib	JAK1	Phase 3	~1–3 days	~60–70%	Nausea, headache, infections	(58, 59, 61, 86, 87)
Upadacitinib	JAK1	Phase 3	~1–2 days	~70–75%	Acne, herpes zoster, hyperlipidemia	(62, 88–94)
Baricitinib	JAK1/JAK2	Phase 3	~1 week	~50–60%	Cytopenia, infections	(69–71, 86, 95)
Ivarmacitinib	JAK1	Phase 3	~3–8 days	~54-66%	Respiratory infection, increased blood creatine phosphokinase, proteinuria, hyperlipidemia, folliculitis, acne	(67, 68, 96, 97)
Topical JAK inhibitors						
Ruxolitinib	JAK1/JAK2	Phase 3	~1 week	~50% (local area)	Local burning, minimal systemic AEs	(72, 98)
Delgocitinib	Pan-JAK	Phase 3	~1–2 weeks	~40–60%	Mild application site irritation	(76–80, 99)
Brepocitinib	TYK2/JAK1	Phase 2	~2–3days	~32-54%	Contact dermatitis, nasopharyngitis	(81)
Tofacitinib	Pan-JAK	Phase 2	Not available	Not available	Dyslipidemia, altered bleeding time	(54, 100, 101)

**Table 2:** Practical considerations for selecting JAK inhibitors in AD.

Patient profile/consideration	Preferred option(s)	Practical advice/cautions	References
Rapid symptom control required (severe itch, sleep loss)	Oral JAK1 inhibitors (Abrocitinib, Upadacitinib)	These agents may provide rapid itch relief, often within 2–3 days. Clinicians should discuss with patients the difference between rapid symptom control and long-term disease management.	(102, 103)
Mild-to-moderate AD or desire to avoid systemic exposure	Topical JAK inhibitors (Ruxolitinib cream, Delgocitinib ointment)	Topical agents are suitable for localized disease and may minimize systemic exposure. They are often used for short-term or intermittent treatment.	(72, 78)
History of recurrent herpes zoster or increased infection risk	Consider biologics (e.g., dupilumab) as first-line; JAK inhibitors may be used cautiously	Vaccination against herpes zoster should be considered prior to initiating systemic JAK inhibitors. Careful monitoring for viral infections is recommended.	(104, 105)
History of thromboembolism or high cardiovascular risk	Prefer biologics or non-JAK systemic therapies	FDA has issued safety warnings regarding potential cardiovascular risks. If JAK inhibitors are required, the lowest effective dose and careful monitoring should be considered.	(106)
Concomitant conjunctivitis or ocular surface disease	JAK inhibitors may be considered as an alternative to dupilumab	Dupilumab therapy has been associated with higher rates of conjunctivitis in some patients; JAK inhibitors may represent an alternative therapeutic option	(107, 108)
Renal impairment	Dose adjustment may be required (e.g., Abrocitinib)	Renal function should be assessed before treatment initiation and dosing should follow product-specific recommendations.	(61)
Pediatric or adolescent patients	Upadacitinib or Abrocitinib (approved in some regions); topical JAK inhibitors for milder cases	Careful monitoring is recommended as long-term safety data in pediatric populations are still evolving.	(60, 62)
Elderly patients (>65 years)	Topical therapy preferred; systemic JAK inhibitors used cautiously	Older patients may have higher baseline risks of infections, cardiovascular disease, and malignancy. Regular laboratory and clinical monitoring is recommended.	(106, 109)
Patient concern about laboratory abnormalities	JAK1-selective inhibitors with regular monitoring	Patients should be informed about potential laboratory changes (lipids, CPK, blood counts), and periodic monitoring should be implemented.	(15, 105)

Efficacy comparisons across clinical trials and network meta-analyses suggest that JAK1-selective inhibitors (abrocitinib and upadacitinib) achieve more rapid and higher rates of skin clearance and itch reduction compared to other agents. For example, in a systematic comparison, abrocitinib 200 mg/day showed the most improvement in the Patient-Oriented Eczema Measure and itch numeric rating scale, while dupilumab had the most impact on quality-of-life scores (86, 110).

Safety profiles differ substantially among JAK inhibitors. Although a pooled meta-analysis found no significant increase in major adverse cardiovascular events or venous thromboembolism compared to placebo or active controls, real-world data suggest a higher incidence of herpes zoster, acne, cytopenia, and hyperlipidemia with oral JAK inhibitors compared to dupilumab (87–92, 95, 98, 111). Furthermore, long-term risks, especially in elderly patients or those with comorbidities, remain under investigation. Conversely, dupilumab is associated with conjunctivitis and ocular surface inflammation, which are less common with JAK inhibitors (70, 99, 107, 108).

The time to clinical response is another practical factor in choosing between JAK inhibitors and biologics. Studies have consistently shown that oral JAK inhibitors achieve itch relief within 2–3 days, a significantly faster onset than dupilumab, which may take 1–2 weeks (93, 94, 102, 103). This rapid effect is particularly valuable in patients with severe pruritus and sleep disturbance.

Overall, JAK inhibitors represent a versatile addition to the therapeutic armamentarium for AD. Their fast onset, oral or topical formulations, and cytokine-modulating breadth provide valuable alternatives to existing biologics. However, careful consideration of individual patient factors, such as age, comorbidities, infection risk, and disease phenotype, is essential in selecting the optimal agent. Age is an important determinant in therapeutic decision-making, as elderly patients often present with higher baseline risks of infections, cardiovascular disease, and malignancy, which may influence the safety profile of systemic JAK inhibitors (112). Similarly, comorbid conditions such as metabolic syndrome, cardiovascular disease, or a history of thromboembolic events should be carefully evaluated when considering systemic JAK inhibition. Infection susceptibility also represents a critical factor, particularly in individuals with a history of recurrent herpes infections or other immunosuppressive conditions, given the increased incidence of herpes zoster and other viral infections associated with JAK inhibitor therapy (113). In addition, disease phenotype and severity may influence treatment choice. Patients with severe pruritus may benefit from the rapid antipruritic effects observed with oral JAK1-selective inhibitors, whereas those with localized or mild disease may be more suitable candidates for topical JAK inhibitors (56, 114). Therefore, individualized assessment of patient characteristics and disease presentation is essential to optimize therapeutic outcomes and minimize potential risks in AD management.

## 5 Controversies and challenges

### 5.1 Debates on the efficacy of JAK/STAT pathway inhibitors

Although JAK inhibitors have demonstrated strong clinical efficacy in many patients with moderate-to-severe AD, their long-term benefits and consistency across diverse populations remain under active debate. The heterogeneity of AD—encompassing variations in endotypes, age of onset, ethnic background, and comorbid allergic conditions—may partly explain the variable therapeutic response to JAK inhibitors (104, 115, 116). While clinical trials have shown rapid improvement of JAK inhibitors in pruritus and skin inflammation, real-world data have also reported cases of incomplete response or early relapse after drug discontinuation (117, 118).

Systematic reviews of JAK inhibitor trials in AD have highlighted methodological issues such as limited sample sizes, short treatment durations, and frequent reliance on placebo comparators rather than active controls (e.g., dupilumab). This lack of head-to-head comparisons complicates interpretation of the true comparative efficacy of JAK inhibitors, especially against long-established biologics. Furthermore, while initial treatment responses are often robust, the sustainability of disease control with JAK inhibitors remains unclear. Long-term extension studies are still ongoing, and concerns persist regarding disease rebound or tachyphylaxis with prolonged use. In particular, whether topical JAK inhibitors maintain efficacy during chronic treatment remains uncertain. These issues necessitate additional long-term, real-world observational data to fully validate the efficacy profile of JAK inhibitors in clinical dermatology practice.

### 5.2 Safety concerns and adverse events

The safety of JAK inhibitors, particularly with systemic administration, is a central concern that limits their widespread use in some patient populations. As JAK inhibitors target intracellular signaling of multiple cytokine families, they can lead to broad immunosuppressive effects, increasing susceptibility to cutaneous and systemic infections. Among the most frequently reported adverse events are herpes simplex, herpes zoster, eczema herpeticum, acne, and folliculitis (109). Notably, the incidence of herpes zoster is higher with JAK inhibitors than with other biologics, likely due to impaired interferon signaling mediated via JAK1/2 inhibition (104).

Beyond infection risk, JAK inhibitors have been associated with hematologic and metabolic abnormalities, including thrombocytopenia, anemia, elevated creatine kinase, and lipid profile changes (15, 105). Routine monitoring of laboratory parameters is recommended, especially in patients receiving high-dose oral therapy. Concerns regarding cardiovascular safety have also emerged, particularly considering data from other immune-mediated conditions such as rheumatoid arthritis. The ORAL Surveillance trial reported increased rates of major adverse cardiovascular events and malignancy in high-risk populations receiving tofacitinib, prompting regulatory agencies to revise safety warnings for JAK inhibitors (106). Although these findings were not replicated in dermatology-specific populations, caution is warranted in patients with baseline cardiovascular risk or a history of smoking.

Lastly, while topical JAK inhibitors are generally better tolerated, long-term data on their cumulative systemic exposure, especially when applied to large surface areas or under occlusion, are still limited. Therefore, clinicians should weigh potential risks versus benefits carefully, particularly in elderly or immunocompromised individuals.

In addition, the interpretation of clinical trial outcomes may be influenced by differences in study design, particularly regarding the use of concomitant topical corticosteroids (TCS) (119). In routine clinical practice, systemic or targeted therapies for AD are frequently combined with topical treatments to optimize disease control. Accordingly, several clinical trials evaluating JAK inhibitors have allowed background TCS therapy, which may better reflect real-world treatment strategies (120–122). In contrast, studies that exclude concomitant topical therapies may underestimate the additive benefits achieved in everyday clinical practice (123, 124). These considerations should be taken into account when comparing treatment efficacy across studies.

### 5.3 Regulatory and ethical considerations

The development, approval, and post-marketing surveillance of JAK inhibitors involve complex regulatory and ethical challenges. Given their pleiotropic effects and potential safety concerns, the U.S. FDA and EMA have issued updated guidance requiring boxed warnings and more stringent risk-benefit assessments for systemic JAK inhibitors in all indications, including AD (125).

Regulatory frameworks now emphasize the importance of long-term data, head-to-head comparisons, and stratified analysis by risk categories (e.g., age, comorbidities). In AD trials, defining appropriate primary endpoints (e.g., EASI-75 vs. itch reduction), ensuring ethnic diversity, and addressing pediatric populations require thoughtful design and scrutiny. From an ethical standpoint, the inclusion of vulnerable populations, particularly children and the elderly, demands special attention. As AD frequently begins in childhood and persists throughout life, long-term safety data are critical. Ethical trial design must ensure comprehensive informed consent that clearly explains the risks of infection, malignancy, and laboratory abnormalities associated with the use of JAK inhibitors (125). Moreover, patient autonomy must be preserved, and alternatives such as topical therapies or biologics should be thoroughly discussed. Privacy concerns also arise with the increasing integration of genetic and biomarker data to predict treatment response. Regulatory guidelines must ensure that such data are stored and used responsibly, in line with international bioethics standards.

Overall, while JAK inhibitors represent a transformative class of therapeutics in AD, optimizing their safe, effective, and ethical deployment remains an ongoing challenge requiring collaboration among clinicians, regulators, and patients.

## 6 Future directions and innovations

### 6.1 Potential for personalized medicine in AD

AD is recognized as a clinically and immunologically heterogeneous disease, with substantial variation in endotypes, biomarker profiles, and treatment responses. This heterogeneity provides a compelling rationale for the advancement of personalized medicine strategies in AD management. The traditional “one-size-fits-all” approach is increasingly being replaced by stratified or individualized therapeutic paradigms based on molecular and clinical profiling.

A promising area of investigation is the use of circulating biomarkers to guide treatment decisions. For instance, thymus and activation-regulated chemokine (TARC/CCL17) has been studied as a marker of Th2-driven inflammation and disease severity in AD (126). However, single biomarker approaches often fail to capture the complexity of the disease. Recent integrative analyses have identified multi-analyte serum panels that provide a more nuanced assessment of disease activity and may help predict therapeutic response, particularly to JAK/STAT-targeting agents (127).

Moreover, unsupervised clustering of patients based on biomarker profiles has revealed distinct molecular endotypes with differing cytokine expression patterns and disease manifestations. In one study, four AD clusters were defined by differences in IL-13, IL-22, and IFN-γ signaling activity, suggesting differential responsiveness to specific targeted therapies (128). Such stratification could allow more rational selection of JAK inhibitors based on dominant pathway activation (e.g., JAK1/STAT6 in Th2-high AD vs. STAT3 in Th17/Th22-biased subtypes).

The cutaneous microbiome also represents a critical and individualized factor in AD. Dysbiosis, particularly the overrepresentation of *S.aureus*, is linked to disease exacerbation. Emerging evidence suggests that certain microbial profiles correlate with treatment resistance or flare frequency. Microbiome-informed therapeutics—including prebiotics, probiotics, or bacteriophage therapy—could be combined with JAK inhibitors to improve barrier function and reduce inflammation in a patient-specific manner (129–132).

### 6.2 Innovations in JAK/STAT pathway research and technology

Ongoing research into the structural biology and regulatory mechanisms of the JAK/STAT pathway is catalyzing the development of more selective, potent, and safer therapeutics. Third-generation JAK inhibitors, including allosteric modulators that bind to the pseudokinase domain, offer enhanced specificity with potentially reduced risk of off-target immune suppression. For example, deucravacitinib, a TYK2 allosteric inhibitor, has shown a favorable safety profile in psoriasis by selectively modulating IL-23 and type I interferon signaling without affecting broader JAK1/2/3 functions (133).

Animal models and gene-editing technologies are also advancing our understanding of JAK/STAT-mediated immunoregulation. Knock-in mice expressing humanized cytokine receptors, such as human IFNAR1/2, enable *in vivo* dissection of human cytokine responses and preclinical evaluation of novel JAK inhibitors (134). These models facilitate mechanistic studies on cytokine redundancy and therapeutic specificity, especially for agents designed to target overlapping signaling cascades.

In parallel, single-cell RNA sequencing and spatial transcriptomics are providing unprecedented resolution into cell-type–specific JAK/STAT activity within diseased skin. This technology has uncovered functionally distinct keratinocyte subsets and immune cell clusters with differential activation of STAT3, STAT6, and STAT1 in AD lesions (135–137). Such insights may inform the design of combination therapies or localized delivery strategies that target the most relevant cellular compartments.

### 6.3 Prospects for JAK/STAT pathway modulation in dermatology

Beyond AD, the therapeutic modulation of the JAK/STAT axis holds considerable promise for a broad spectrum of dermatologic disorders, including psoriasis, alopecia areata, vitiligo, and hidradenitis suppurativa. The success of TYK2 inhibition in psoriasis has exemplified the feasibility of pathway-selective intervention in immune-mediated skin diseases (137). In the context of AD, future research is expected to focus on long-term safety evaluation, dose optimization, and treatment sequencing. Questions remain regarding the optimal duration of JAK inhibitor therapy, the safety of continuous versus intermittent dosing, and the potential benefits of combining JAK inhibitors with other biologics (e.g., dupilumab) or topical anti-inflammatory agents. Additionally, the development of advanced drug delivery systems may improve the therapeutic index of JAK inhibitors. For example, gold nanoparticle–conjugated ruxolitinib has been investigated for topical delivery, demonstrating enhanced skin penetration and lower systemic absorption in preclinical alopecia models (138). Liposomal and microneedle-based systems are also under exploration to facilitate targeted epidermal delivery while minimizing systemic exposure, particularly in pediatric and elderly populations. Altogether, the integration of precision medicine tools, advanced molecular profiling, and drug delivery innovations is expected to redefine the landscape of JAK/STAT-targeted therapies in dermatology.

## 7 Conclusion

The JAK/STAT signaling pathway has emerged as a central mechanism in the pathogenesis and therapeutic targeting of AD. It orchestrates the effects of numerous pro-inflammatory and pruritogenic cytokines involved in both acute and chronic phases of the disease. The advent of selective JAK inhibitors—both oral and topical—has broadened the treatment landscape, offering rapid symptom control and improved patient outcomes. However, concerns remain regarding their long-term safety, particularly in systemic formulations, necessitating vigilant risk-benefit evaluation and personalized therapeutic strategies. Future research is poised to integrate molecular biomarkers, genomic profiling, and skin microbiome signatures into the clinical decision-making process. The development of next-generation JAK inhibitors with improved specificity, alongside advanced drug delivery systems, holds promise for enhancing efficacy while minimizing adverse effects. Ultimately, a precision medicine approach—rooted in mechanistic understanding of JAK/STAT signaling and tailored to individual patient profiles—may redefine the standard of care for AD and other chronic inflammatory skin diseases.

## References

[1] Schuler C Billi AC Maverakis E Tsoi LC Gudjonsson JE . Novel insights into atopic dermatitis. J Allergy Clin Immunol. (2023) 151:1145–54. doi: 10.1016/j.jaci.2022.10.023, PMID: 36428114 PMC10164702

[2] Guttman-Yassky E Renert-Yuval Y Brunner PM . Atopic dermatitis. Lancet. (2025) 405:583–96. doi: 10.1016/s0140-6736(24)02519-4, PMID: 39955121

[3] Sroka-Tomaszewska J Trzeciak M . Molecular mechanisms of atopic dermatitis pathogenesis. Int J Mol Sci. (2021) 22:4130–45. doi: 10.3390/ijms22084130, PMID: 33923629 PMC8074061

[4] Afshari M Kolackova M Rosecka M Čelakovská J Krejsek J . Unraveling the skin; a comprehensive review of atopic dermatitis, current understanding, and approaches. Front Immunol. (2024) 15:1361005. doi: 10.3389/fimmu.2024.1361005, PMID: 38500882 PMC10944924

[5] Rajkumar J Chandan N Lio P Shi V . The skin barrier and moisturization: function, disruption, and mechanisms of repair. Skin Pharmacol Physiol. (2023) 36:174–85. doi: 10.1159/000534136, PMID: 37717558

[6] Kupschke E Schenk M . The myeloid switch: immune drivers in atopic dermatitis - roles in pathogenesis and emerging therapeutic targeting. Front Immunol. (2025) 16:1608338. doi: 10.3389/fimmu.2025.1608338, PMID: 40661941 PMC12256259

[7] Gandhi NA Bennett BL Graham NM Pirozzi G Stahl N Yancopoulos GD . Targeting key proximal drivers of type 2 inflammation in disease. Nat Rev Drug Discov. (2016) 15:35–50. doi: 10.1038/nrd4624, PMID: 26471366

[8] Wynn TA . Type 2 cytokines: mechanisms and therapeutic strategies. Nat Rev Immunol. (2015) 15:271–82. doi: 10.1038/nri3831, PMID: 25882242

[9] Kim HB Alexander H Um JY Chung BY Park CW Flohr C . Skin microbiome dynamics in atopic dermatitis: understanding host-microbiome interactions. Allergy Asthma Immunol Res. (2025) 17:165–80. doi: 10.4168/aair.2025.17.2.165, PMID: 40204503 PMC11982640

[10] D’Auria E Indolfi C Acunzo M Dinardo G Comberiati P Peroni D . Biologics and small molecules: the re-evolution in the treatment of atopic dermatitis in children and adolescents. Current state of the art and future perspectives. Expert Rev Clin Immunol. (2025) 21:493–505. doi: 10.1080/1744666X.2025.2452247, PMID: 39810497

[11] Guttman-Yassky E Irvine AD Brunner PM Kim BS Boguniewicz M Parmentier J . The role of Janus kinase signaling in the pathology of atopic dermatitis. J Allergy Clin Immunol. (2023) 152:1394–404. doi: 10.1016/j.jaci.2023.07.010, PMID: 37536511

[12] Rah B Rather RA Bhat GR Baba AB Mushtaq I Farooq M . JAK/STAT signaling: molecular targets, therapeutic opportunities, and limitations of targeted inhibitions in solid Malignancies. Front Pharmacol. (2022) 13:821344. doi: 10.3389/fphar.2022.821344, PMID: 35401182 PMC8987160

[13] Huang IH Chung WH Wu PC Chen CB . JAK-STAT signaling pathway in the pathogenesis of atopic dermatitis: An updated review. Front Immunol. (2022) 13:1068260. doi: 10.3389/fimmu.2022.1068260, PMID: 36569854 PMC9773077

[14] Iznardo H Roé E Serra-Baldrich E Puig L . Efficacy and safety of JAK1 inhibitor abrocitinib in atopic dermatitis. Pharmaceutics. (2023) 15:385–400. doi: 10.3390/pharmaceutics15020385, PMID: 36839707 PMC9960033

[15] Taylor PC Choy E Baraliakos X Szekanecz Z Xavier RM Isaacs JD . Differential properties of Janus kinase inhibitors in the treatment of immune-mediated inflammatory diseases. Rheumatol (Oxford). (2024) 63:298–308. doi: 10.1093/rheumatology/kead448, PMID: 37624925 PMC10836981

[16] Xue C Yao Q Gu X Shi Q Yuan X Chu Q . Evolving cognition of the JAK-STAT signaling pathway: autoimmune disorders and cancer. Signal Transduct Target Ther. (2023) 8:204–27. doi: 10.1038/s41392-023-01468-7, PMID: 37208335 PMC10196327

[17] Hu Q Bian Q Rong D Wang L Song J Huang HS . JAK/STAT pathway: Extracellular signals, diseases, immunity, and therapeutic regimens. Front Bioeng Biotechnol. (2023) 11:1110765. doi: 10.3389/fbioe.2023.1110765, PMID: 36911202 PMC9995824

[18] Wang L Xian YF Loo SKF Ip SP Yang W Chan WY . Baicalin ameliorates 2, 4-dinitrochlorobenzene-induced atopic dermatitis-like skin lesions in mice through modulating skin barrier function, gut microbiota and JAK/STAT pathway. Bioorg Chem. (2022) 119:105538. doi: 10.1016/j.bioorg.2021.105538, PMID: 34929516

[19] Kamata M Tada Y . Optimal use of jak inhibitors and biologics for atopic dermatitis on the basis of the current evidence. JID Innov. (2023) 3:100195. doi: 10.1016/j.xjidi.2023.100195, PMID: 37180768 PMC10173000

[20] Zheng Y Zhang J Guo T Cao J Wang L Zhang J . Canine interleukin-31 binds directly to OSMRβ with higher binding affinity than to IL-31RA. 3 Biotech. (2023) 13:302–12. doi: 10.1007/s13205-023-03724-7, PMID: 37588794 PMC10425310

[21] Mishra B Ivashkiv LB . Interferons and epigenetic mechanisms in training, priming and tolerance of monocytes and hematopoietic progenitors. Immunol Rev. (2024) 323:257–75. doi: 10.1111/imr.13330, PMID: 38567833 PMC11102283

[22] Ivashkiv LB . IFNγ: signalling, epigenetics and roles in immunity, metabolism, disease and cancer immunotherapy. Nat Rev Immunol. (2018) 18:545–58. doi: 10.1038/s41577-018-0029-z, PMID: 29921905 PMC6340644

[23] Runtsch MC Angiari S Hooftman A Wadhwa R Zhang Y Zheng Y . Itaconate and itaconate derivatives target JAK1 to suppress alternative activation of macrophages. Cell Metab. (2022) 34:487–501.e8. doi: 10.1016/j.cmet.2022.02.002, PMID: 35235776

[24] Ouyang W Löhning M Gao Z Assenmacher M Ranganath S Radbruch A . Stat6-independent GATA-3 autoactivation directs IL-4-independent Th2 development and commitment. Immunity. (2000) 12:27–37. doi: 10.1016/s1074-7613(00)80156-9, PMID: 10661403

[25] Sugaya M . The role of th17-related cytokines in atopic dermatitis. Int J Mol Sci. (2020) 21:1314–25. doi: 10.3390/ijms21041314, PMID: 32075269 PMC7072946

[26] Damasceno LEA Prado DS Veras FP Fonseca MM Toller-Kawahisa JE Rosa MH . PKM2 promotes Th17 cell differentiation and autoimmune inflammation by fine-tuning STAT3 activation. J Exp Med. (2020) 217:e20190613. doi: 10.1084/jem.20190613, PMID: 32697823 PMC7537396

[27] Chiricozzi A Maurelli M Calabrese L Peris K Girolomoni G . Overview of atopic dermatitis in different ethnic groups. J Clin Med. (2023) 12:2701–13. doi: 10.3390/jcm12072701, PMID: 37048783 PMC10095524

[28] Bauché D Joyce-Shaikh B Fong J Villarino AV Ku KS Jain R . IL-23 and IL-2 activation of STAT5 is required for optimal IL-22 production in ILC3s during colitis. Sci Immunol. (2020) 5:eaav1080. doi: 10.1126/sciimmunol.aav1080, PMID: 32332067

[29] Yoo SA Kim KC Lee JH . Efficacy and potential mechanisms of naringin in atopic dermatitis. Int J Mol Sci. (2024) 25:11064. doi: 10.3390/ijms252011064, PMID: 39456844 PMC11507659

[30] Banzon TM Kelly MS Bartnikas LM Sheehan WJ Cunningham A Harb H . Atopic dermatitis mediates the association between an IL4RA variant and food allergy in school-aged children. J Allergy Clin Immunol Pract. (2022) 10:2117–2124.e4. doi: 10.1016/j.jaip.2022.04.042, PMID: 35589010 PMC9811396

[31] Gaceja KV Ancheta ZFR Buna ACA Clarencio SMS Garrido MAR Ramos JDA . Association of interleukin-13 gene single nucleotide polymorphism rs1800925 with allergic asthma in Asian population: A meta-analysis. Asia Pac Allergy. (2023) 13:148–57. doi: 10.5415/apallergy.0000000000000119, PMID: 38094093 PMC10715742

[32] Tanaka N Koido M Suzuki A Otomo N Suetsugu H Kochi Y . Eight novel susceptibility loci and putative causal variants in atopic dermatitis. J Allergy Clin Immunol. (2021) 148:1293–306. doi: 10.1016/j.jaci.2021.04.019, PMID: 34116867

[33] Nam YK Jin SC Kim MH Choi Y Lee YB Yang WM . Banhahubak-tang tablet, a standardized medicine attenuates allergic asthma via inhibition of janus kinase 1 (JAK1)/signal transducer and activator of transcription 6 (STAT6) signal pathway. Molecules. (2020) 25:2206–21. doi: 10.3390/molecules25092206, PMID: 32397290 PMC7248972

[34] Dendrou CA Cortes A Shipman L Evans HG Attfield KE Jostins L . Resolving TYK2 locus genotype-to-phenotype differences in autoimmunity. Sci Transl Med. (2016) 8:363ra149. doi: 10.1126/scitranslmed.aag1974, PMID: 27807284 PMC5737835

[35] Banerjee S Biehl A Gadina M Hasni S Schwartz DM . JAK-STAT signaling as a target for inflammatory and autoimmune diseases: current and future prospects. Drugs. (2017) 77:521–46. doi: 10.1007/s40265-017-0701-9, PMID: 28255960 PMC7102286

[36] Pardanani A Fridley BL Lasho TL Gilliland DG Tefferi A . Host genetic variation contributes to phenotypic diversity in myeloproliferative disorders. Blood. (2008) 111:2785–9. doi: 10.1182/blood-2007-06-095703, PMID: 18006699

[37] Kumar S Sharma R Komal K Kumar D Ghosh R Kumar M . Unlocking the molecular pathway of atopic dermatitis: journey so far and roads ahead. Inflammopharmacology. (2025) 33:5203–34. doi: 10.1007/s10787-025-01900-0, PMID: 40824373

[38] Meesters LD Roubroeks JAY Gerritsen A Velthuijs N Klijnhout JA Laberthonnière C . Dissecting key contributions of T(H)2 and T(H)17 cytokines to atopic dermatitis pathophysiology. J Allergy Clin Immunol. (2025) 156:690–704. doi: 10.1016/j.jaci.2025.05.007, PMID: 40409379

[39] Tsoi LC Rodriguez E Degenhardt F Baurecht H Wehkamp U Volks N . Atopic dermatitis is an IL-13-dominant disease with greater molecular heterogeneity compared to psoriasis. J Invest Dermatol. (2019) 139:1480–9. doi: 10.1016/j.jid.2018.12.018, PMID: 30641038 PMC6711380

[40] Kisich KO Carspecken CW Fiéve S Boguniewicz M Leung DY . Defective killing of Staphylococcus aureus in atopic dermatitis is associated with reduced mobilization of human beta-defensin-3. J Allergy Clin Immunol. (2008) 122:62–8. doi: 10.1016/j.jaci.2008.04.022, PMID: 18538383

[41] Czimmerer Z Daniel B Horvath A Rückerl D Nagy G Kiss M . The transcription factor STAT6 mediates direct repression of inflammatory enhancers and limits activation of alternatively polarized macrophages. Immunity. (2018) 48:75–90.e6. doi: 10.1016/j.immuni.2017.12.010, PMID: 29343442 PMC5772169

[42] Di Salvo E Allegra A Casciaro M Gangemi S . IL-31, itch and hematological Malignancies. Clin Mol Allergy. (2021) 19:8–13. doi: 10.1186/s12948-021-00148-7, PMID: 34118946 PMC8199420

[43] Kopalli SR Annamneedi VP Koppula S . Potential natural biomolecules targeting JAK/STAT/SOCS signaling in the management of atopic dermatitis. Molecules. (2022) 27:4660. doi: 10.3390/molecules27144660, PMID: 35889539 PMC9319717

[44] Bao L Zhang H Chan LS . The involvement of the JAK-STAT signaling pathway in chronic inflammatory skin disease atopic dermatitis. Jakstat. (2013) 2:e24137. doi: 10.4161/jkst.24137, PMID: 24069552 PMC3772104

[45] Gittler JK Shemer A Suárez-Fariñas M Fuentes-Duculan J Gulewicz KJ Wang CQ . Progressive activation of T(H)2/T(H)22 cytokines and selective epidermal proteins characterizes acute and chronic atopic dermatitis. J Allergy Clin Immunol. (2012) 130:1344–54. doi: 10.1016/j.jaci.2012.07.012, PMID: 22951056 PMC3991245

[46] Zhang X Wang J Zhu J Liang Y . Downregulation of SHANK-associated RH domain-interacting protein elevates interleukin-33 expression by stimulating the Janus kinase 2/signal transducer and activator of transcription signalling pathway in HaCaT cells. Clin Exp Dermatol. (2021) 46:880–7. doi: 10.1111/ced.14591, PMID: 33548083

[47] Deng Y Leib N Schnautz S Benfadal S Oldenburg J Bieber T . Langerhans cell modulation in atopic dermatitis is TLR2/SOCS1-dependent and JAK inhibitor-sensitive. Allergy. (2025) 80:2586–99. doi: 10.1111/all.16641, PMID: 40631910 PMC12444830

[48] Renert-Yuval Y Thyssen JP Bissonnette R Bieber T Kabashima K Hijnen D . Biomarkers in atopic dermatitis-a review on behalf of the International Eczema Council. J Allergy Clin Immunol. (2021) 147:1174–1190.e1. doi: 10.1016/j.jaci.2021.01.013, PMID: 33516871 PMC11304440

[49] Nygaard U Hvid M Johansen C Buchner M Fölster-Holst R Deleuran M . TSLP, IL-31, IL-33 and sST2 are new biomarkers in endophenotypic profiling of adult and childhood atopic dermatitis. J Eur Acad Dermatol Venereol. (2016) 30:1930–8. doi: 10.1111/jdv.13679, PMID: 27152943

[50] Qiao W Xie T Lu J Jia T Kaku K . Identification of potential hub genes associated with atopic dermatitis-like recombinant human epidermal model using integrated transcriptomic and proteomic analysis. Biomol BioMed. (2024) 24:89–100. doi: 10.17305/bb.2023.9439, PMID: 37540585 PMC10787623

[51] Yu L Li L . Potential biomarkers of atopic dermatitis. Front Med (Lausanne). (2022) 9:1028694. doi: 10.3389/fmed.2022.1028694, PMID: 36465933 PMC9712451

[52] Shao J Xie Z Ye Z Chen G Wang Y Zhang L . Human umbilical cord mesenchymal stem cell therapy for atopic dermatitis through inhibition of neutrophil chemotaxis. Stem Cell Res Ther. (2025) 16:243–56. doi: 10.1186/s13287-025-04349-8, PMID: 40369633 PMC12079922

[53] Marega LF Sabino JS Pedroni MV Teocchi M Lanaro C de Albuquerque DM . Phenotypes of STAT3 gain-of-function variant related to disruptive regulation of CXCL8/STAT3, KIT/STAT3, and IL-2/CD25/Treg axes. Immunol Res. (2021) 69:445–56. doi: 10.1007/s12026-021-09225-0, PMID: 34390446

[54] Faletti L Ehl S Heeg M . Germline STAT3 gain-of-function mutations in primary immunodeficiency: Impact on the cellular and clinical phenotype. BioMed J. (2021) 44:412–21. doi: 10.1016/j.bj.2021.03.003, PMID: 34366294 PMC8514798

[55] Vogel TP Leiding JW Cooper MA Forbes Satter LR . STAT3 gain-of-function syndrome. Front Pediatr. (2022) 10:770077. doi: 10.3389/fped.2022.770077, PMID: 36843887 PMC9948021

[56] Chovatiya R Paller AS . JAK inhibitors in the treatment of atopic dermatitis. J Allergy Clin Immunol. (2021) 148:927–40. doi: 10.1016/j.jaci.2021.08.009, PMID: 34437922 PMC10166130

[57] Dhar S De A Sarda A Godse K Lahiri K . Real-world efficacy and safety of oral tofacitinib in patients with refractory moderate to severe atopic dermatitis: A multicenter retrospective study. Indian J Dermatol. (2024) 69:292–5. doi: 10.4103/ijd.ijd_843_22, PMID: 39296687 PMC11407563

[58] Simpson EL Sinclair R Forman S Wollenberg A Aschoff R Cork M . Efficacy and safety of abrocitinib in adults and adolescents with moderate-to-severe atopic dermatitis (JADE MONO-1): a multicentre, double-blind, randomised, placebo-controlled, phase 3 trial. Lancet. (2020) 396:255–66. doi: 10.1016/s0140-6736(20)30732-7, PMID: 32711801

[59] Silverberg JI Simpson EL Thyssen JP Gooderham M Chan G Feeney C . Efficacy and safety of abrocitinib in patients with moderate-to-severe atopic dermatitis: A randomized clinical trial. JAMA Dermatol. (2020) 156:863–73. doi: 10.1001/jamadermatol.2020.1406, PMID: 32492087 PMC7271424

[60] Shi VY Bhutani T Fonacier L Deleuran M Shumack S Valdez H . Phase 3 efficacy and safety of abrocitinib in adults with moderate-to-severe atopic dermatitis after switching from dupilumab (JADE EXTEND). J Am Acad Dermatol. (2022) 87:351–8. doi: 10.1016/j.jaad.2022.04.009, PMID: 35439608

[61] Gooderham MJ Forman SB Bissonnette R Beebe JS Zhang W Banfield C . Efficacy and safety of oral janus kinase 1 inhibitor abrocitinib for patients with atopic dermatitis: A phase 2 randomized clinical trial. JAMA Dermatol. (2019) 155:1371–9. doi: 10.1001/jamadermatol.2019.2855, PMID: 31577341 PMC6777226

[62] Paller AS Ladizinski B Mendes-Bastos P Siegfried E Soong W Prajapati VH . Efficacy and safety of upadacitinib treatment in adolescents with moderate-to-severe atopic dermatitis: analysis of the measure up 1, measure up 2, and AD up randomized clinical trials. JAMA Dermatol. (2023) 159:526–35. doi: 10.1001/jamadermatol.2023.0391, PMID: 37043227 PMC10099102

[63] Chu AWL Wong MM Rayner DG Guyatt GH Díaz Martinez JP Ceccacci R . Systemic treatments for atopic dermatitis (eczema): Systematic review and network meta-analysis of randomized trials. J Allergy Clin Immunol. (2023) 152:1470–92. doi: 10.1016/j.jaci.2023.08.029, PMID: 37678577

[64] Yusof MHM Jinih M . Infection risk profiles of upadacitinib in patient with rheumatoid arthritis: A meta-analysis of randomized clinical trials. J Clin Rheumatol Immunol. (2024) 24:69–70. doi: 10.1142/S2661341724740493, PMID: 40951326

[65] Choy E Mcinnes I Cush J Aelion J Rigby W Song Y . THU0195INCIDENCE and risk of venous thromboembolic events among patients with rheumatoid arthritis enrolled in the upadacitinib select clinical trial program. Ann Rheumatic Dis. (2020) 79:2. doi: 10.1136/annrheumdis-2020-eular.2897, PMID: 41802225

[66] Runde J Ryan K Hirst J Lebowitz J Chen W Brown J . Upadacitinib is associated with clinical response and steroid-free remission for children and adolescents with inflammatory bowel disease. J Pediatr Gastroenterol Nutr. (2025) 80:133–40. doi: 10.1002/jpn3.12408, PMID: 39538977

[67] Zhao Y Gooderham M Yang B Wu J Wu L Loo WJ . Ivarmacitinib for moderate to severe atopic dermatitis in adults and adolescents: A phase 3 randomized clinical trial. JAMA Dermatol. (2025) 161:688–97. doi: 10.1001/jamadermatol.2025.0982, PMID: 40305055 PMC12044538

[68] Keam SJ . Ivarmacitinib sulfate: first approval. Drugs. (2025) 85:1163–70. doi: 10.1007/s40265-025-02202-z, PMID: 40542222

[69] Kamata M . Real-world effectiveness and safety of baricitinib including its effect on biomarkers and laboratory data in Japanese adult patients with atopic dermatitis: a single-center retrospective study. J Cutaneous Immunol Allergy. (2024) 7:12455. doi: 10.3389/jcia.2024.12455, PMID: 41869664

[70] Wollenberg A Ikeda M Chu CY Eichenfield LF Seyger MMB Prakash A . Longer-term safety and efficacy of baricitinib for atopic dermatitis in pediatric patients 2 to <18 years old: a randomized clinical trial of extended treatment to 3.6 years. J Dermatolog Treat. (2024) 35:2411834. doi: 10.1080/09546634.2024.2411834, PMID: 39522957

[71] Guttman-Yassky E Silverberg JI Nemoto O Forman SB Wilke A Prescilla R . Baricitinib in adult patients with moderate-to-severe atopic dermatitis: A phase 2 parallel, double-blinded, randomized placebo-controlled multiple-dose study. J Am Acad Dermatol. (2019) 80:913–21. doi: 10.1016/j.jaad.2018.01.018, PMID: 29410014

[72] Gold LS Bissonnette R Forman S Zaenglein A Kuo YT Angel B . A maximum-use trial of ruxolitinib cream in children aged 2–11 years with moderate to severe atopic dermatitis. Am J Clin Dermatol. (2025) 26:275–89. doi: 10.1007/s40257-024-00909-5, PMID: 39760983 PMC11850462

[73] Eichenfield LF Stein Gold LF Simpson EL Zaenglein AL Armstrong AW Tollefson MM . Efficacy and safety of ruxolitinib cream in children aged 2 to 11 years with atopic dermatitis: Results from TRuE-AD3, a phase 3, randomized double-blind study. J Am Acad Dermatol. (2025) 93:689–98. doi: 10.1016/j.jaad.2025.05.1385, PMID: 40378883

[74] Simpson EL Augustin M Thaçi D Misery L Armstrong AW Blauvelt A . Ruxolitinib cream monotherapy improved symptoms and quality of life in adults and adolescents with mild-to-moderate atopic dermatitis: patient-reported outcomes from two phase III studies. Am J Clin Dermatol. (2025) 26:121–37. doi: 10.1007/s40257-024-00901-z, PMID: 39546129 PMC11742460

[75] Gong X Chen X Kuligowski ME Liu X Liu X Cimino E . Pharmacokinetics of ruxolitinib in patients with atopic dermatitis treated with ruxolitinib cream: data from phase II and III studies. Am J Clin Dermatol. (2021) 22:555–66. doi: 10.1007/s40257-021-00610-x, PMID: 33982267 PMC8200345

[76] Fukuie T Toyama H Tanaka M Ohashi-Doi K Kabashima K . *Post-hoc* safety/efficacy analyses from pediatric delgocitinib atopic dermatitis trials. Pediatr Int. (2024) 66:e15798. doi: 10.1111/ped.15798, PMID: 39373522 PMC11580369

[77] Igarashi A Nakagawa H . Safety and efficacy of delgocitinib ointment in adult patients with atopic dermatitis based on disease severity. J Japan Organ Clin Dermatologists. (2022) 39:593–9. doi: 10.3812/jocd.39.593

[78] Abe M Iizuka H Nemoto-Hasebe I Nemoto O Toyama H Ohashi-Doi K . Clinical effect of delgocitinib 0.5% ointment on atopic dermatitis eczema intensity and skin barrier function. J Cutaneous Immunol Allergy. (2021) 5:38–46. doi: 10.1002/cia2.12213, PMID: 41859965

[79] Worm M Thyssen JP Schliemann S Bauer A Shi VY Ehst B . The pan-JAK inhibitor delgocitinib in a cream formulation demonstrates dose response in chronic hand eczema in a 16-week randomized phase IIb trial. Br J Dermatol. (2022) 187:42–51. doi: 10.1111/bjd.21037, PMID: 35084738 PMC9350381

[80] Nakagawa H Nemoto O Yamada H Nagata T Ninomiya N . Phase 1 studies to assess the safety, tolerability and pharmacokinetics of JTE-052 (a novel Janus kinase inhibitor) ointment in Japanese healthy volunteers and patients with atopic dermatitis. J Dermatol. (2018) 45:701–9. doi: 10.1111/1346-8138.14322, PMID: 29665062 PMC6001687

[81] Landis MN Arya M Smith S Draelos Z Usdan L Tarabar S . Efficacy and safety of topical brepocitinib for the treatment of mild-to-moderate atopic dermatitis: a phase IIb, randomized, double-blind, vehicle-controlled, dose-ranging and parallel-group study. Br J Dermatol. (2022) 187:878–87. doi: 10.1111/bjd.21826, PMID: 35986699 PMC10092158

[82] Pavel AB Song T Kim HJ Del Duca E Krueger JG Dubin C . Oral Janus kinase/SYK inhibition (ASN002) suppresses inflammation and improves epidermal barrier markers in patients with atopic dermatitis. J Allergy Clin Immunol. (2019) 144:1011–24. doi: 10.1016/j.jaci.2019.07.013, PMID: 31356921

[83] Bissonnette R Maari C Forman S Bhatia N Lee M Fowler J . The oral Janus kinase/spleen tyrosine kinase inhibitor ASN002 demonstrates efficacy and improves associated systemic inflammation in patients with moderate-to-severe atopic dermatitis: results from a randomized double-blind placebo-controlled study. Br J Dermatol. (2019) 181:733–42. doi: 10.1111/bjd.17932, PMID: 30919407 PMC6850605

[84] Piscitelli SC Pavel AB McHale K Jett JE Collins J Gillmor D . A phase 1b, randomized, single-center trial of topical cerdulatinib (DMVT-502) in patients with mild-to-moderate atopic dermatitis. J Invest Dermatol. (2021) 141:1847–51. doi: 10.1016/j.jid.2020.11.031, PMID: 33493530

[85] Calabrese L D’Onghia M Lazzeri L Rubegni G Cinotti E . Blocking the IL-4/IL-13 axis versus the JAK/STAT pathway in atopic dermatitis: how can we choose? J Pers Med. (2024) 14:775–90. doi: 10.3390/jpm14070775, PMID: 39064029 PMC11278138

[86] Bieber T Simpson EL Silverberg JI Thaçi D Paul C Pink AE . Abrocitinib versus placebo or dupilumab for atopic dermatitis. N Engl J Med. (2021) 384:1101–12. doi: 10.1056/NEJMoa2019380, PMID: 33761207

[87] Simpson EL Silverberg JI Nosbaum A Winthrop K Guttman-Yassky E Hoffmeister KM . Integrated safety update of abrocitinib in 3802 patients with moderate-to-severe atopic dermatitis: data from more than 5200 patient-years with up to 4 years of exposure. Am J Clin Dermatol. (2024) 25:639–54. doi: 10.1007/s40257-024-00869-w, PMID: 38888681 PMC11193687

[88] Blauvelt A Teixeira HD Simpson EL Costanzo A De Bruin-Weller M Barbarot S . Efficacy and safety of upadacitinib vs dupilumab in adults with moderate-to-severe atopic dermatitis: A randomized clinical trial. JAMA Dermatol. (2021) 157:1047–55. doi: 10.1001/jamadermatol.2021.3023, PMID: 34347860 PMC8340015

[89] Blauvelt A Ladizinski B Prajapati VH Laquer V Fischer A Eisman S . Efficacy and safety of switching from dupilumab to upadacitinib versus continuous upadacitinib in moderate-to-severe atopic dermatitis: Results from an open-label extension of the phase 3, randomized, controlled trial (Heads Up). J Am Acad Dermatol. (2023) 89:478–85. doi: 10.1016/j.jaad.2023.05.033, PMID: 37230366

[90] Reich K Teixeira HD de Bruin-Weller M Bieber T Soong W Kabashima K . Safety and efficacy of upadacitinib in combination with topical corticosteroids in adolescents and adults with moderate-to-severe atopic dermatitis (AD Up): results from a randomised, double-blind, placebo-controlled, phase 3 trial. Lancet. (2021) 397:2169–81. doi: 10.1016/s0140-6736(21)00589-4, PMID: 34023009

[91] Guttman-Yassky E Thaçi D Pangan AL Hong HC Papp KA Reich K . Upadacitinib in adults with moderate to severe atopic dermatitis: 16-week results from a randomized, placebo-controlled trial. J Allergy Clin Immunol. (2020) 145:877–84. doi: 10.1016/j.jaci.2019.11.025, PMID: 31786154

[92] Silverberg JI de Bruin-Weller M Bieber T Soong W Kabashima K Costanzo A . Upadacitinib plus topical corticosteroids in atopic dermatitis: Week 52 AD Up study results. J Allergy Clin Immunol. (2022) 149:977–87. doi: 10.1016/j.jaci.2021.07.036, PMID: 34403658

[93] Simpson EL Prajapati VH Leshem YA Chovatiya R de Bruin-Weller MS Ständer S . Upadacitinib rapidly improves patient-reported outcomes in atopic dermatitis: 16-week results from phase 3 clinical trials (Measure up 1 and 2). Dermatol Ther (Heidelb). (2024) 14:1127–44. doi: 10.1007/s13555-024-01157-5, PMID: 38696027 PMC11116320

[94] Silverberg JI Bunick CG Hong HC Mendes-Bastos P Stein Gold L Costanzo A . Efficacy and safety of upadacitinib versus dupilumab in adults and adolescents with moderate-to-severe atopic dermatitis: week 16 results of an open-label randomized efficacy assessor-blinded head-to-head phase IIIb/IV study (Level Up). Br J Dermatol. (2024) 192:36–45. doi: 10.1093/bjd/ljae404, PMID: 39438067

[95] Torrelo A Rewerska B Galimberti M Paller A Yang CY Prakash A . Efficacy and safety of baricitinib in combination with topical corticosteroids in paediatric patients with moderate-to-severe atopic dermatitis with an inadequate response to topical corticosteroids: results from a phase III, randomized, double-blind, placebo-controlled study (BREEZE-AD PEDS). Br J Dermatol. (2023) 189:23–32. doi: 10.1093/bjd/ljad096, PMID: 36999560

[96] Zhao Y Zhang L Ding Y Tao X Ji C Dong X . Efficacy and safety of SHR0302, a highly selective janus kinase 1 inhibitor, in patients with moderate to severe atopic dermatitis: A phase II randomized clinical trial. Am J Clin Dermatol. (2021) 22:877–89. doi: 10.1007/s40257-021-00627-2, PMID: 34374027 PMC8351769

[97] Li B Li N Goh AH Cao H Irwin MW Tong X . A phase I study to evaluate the safety and pharmacokinetics of SHR0302 base ointment in healthy adult volunteers. Skin Pharmacol Physiol. (2023) 36:76–86. doi: 10.1159/000528739, PMID: 36580897

[98] Simpson EL Kircik L Blauvelt A Kallender H Sturm D Wang M . Ruxolitinib cream in adolescents/adults with atopic dermatitis meeting severity thresholds for systemic therapy: exploratory analysis of pooled results from two phase 3 studies. Dermatol Ther (Heidelb). (2024) 14:2139–51. doi: 10.1007/s13555-024-01219-8, PMID: 38995504 PMC11333780

[99] Nakagawa H Nemoto O Igarashi A Saeki H Kaino H Nagata T . Delgocitinib ointment, a topical Janus kinase inhibitor, in adult patients with moderate to severe atopic dermatitis: A phase 3, randomized, double-blind, vehicle-controlled study and an open-label, long-term extension study. J Am Acad Dermatol. (2020) 82:823–31. doi: 10.1016/j.jaad.2019.12.015, PMID: 32029304

[100] Levy LL Urban J King BA . Treatment of recalcitrant atopic dermatitis with the oral Janus kinase inhibitor tofacitinib citrate. J Am Acad Dermatol. (2015) 73:395–9. doi: 10.1016/j.jaad.2015.06.045, PMID: 26194706

[101] Bissonnette R Papp KA Poulin Y Gooderham M Raman M Mallbris L . Topical tofacitinib for atopic dermatitis: a phase IIa randomized trial. Br J Dermatol. (2016) 175:902–11. doi: 10.1111/bjd.14871, PMID: 27423107

[102] Ständer S Kwatra SG Silverberg JI Simpson EL Thyssen JP Yosipovitch G . Early itch response with abrocitinib is associated with later efficacy outcomes in patients with moderate-to-severe atopic dermatitis: subgroup analysis of the randomized phase III JADE COMPARE trial. Am J Clin Dermatol. (2023) 24:97–107. doi: 10.1007/s40257-022-00738-4, PMID: 36512175 PMC10032219

[103] Guttman-Yassky E Teixeira HD Simpson EL Papp KA Pangan AL Blauvelt A . Once-daily upadacitinib versus placebo in adolescents and adults with moderate-to-severe atopic dermatitis (Measure Up 1 and Measure Up 2): results from two replicate double-blind, randomised controlled phase 3 trials. Lancet. (2021) 397:2151–68. doi: 10.1016/s0140-6736(21)00588-2, PMID: 34023008

[104] Mu Z Zhao Y Liu X Chang C Zhang J . Molecular biology of atopic dermatitis. Clin Rev Allergy Immunol. (2014) 47:193–218. doi: 10.1007/s12016-014-8415-1, PMID: 24715253

[105] Sechi A Song J Dell’Antonia M Heidemeyer K Piraccini BM Starace M . Adverse events in patients treated with Jak-inhibitors for alopecia areata: A systematic review. J Eur Acad Dermatol Venereol. (2023) 4:1535–46. doi: 10.1111/jdv.19090, PMID: 37013725

[106] Ytterberg SR Bhatt DL Mikuls TR Koch GG Fleischmann R Rivas JL . Cardiovascular and cancer risk with tofacitinib in rheumatoid arthritis. New Engl J Med. (2022) 386:1766–8. doi: 10.1056/NEJMc2202778, PMID: 35081280

[107] Pradhan SP Sadiq SN Cartes C Babakinejad P Ball S Reynolds NJ . Dupilumab induced ocular surface disease: A prospective case series. Eur J Ophthalmol. (2024) 34:691–9. doi: 10.1177/11206721231199155, PMID: 37644849

[108] Lamberg O Pandher K Troost JP Lim HW . Long-term adverse event risks of oral JAK inhibitors versus immunomodulators: a literature review. Arch Dermatol Res. (2024) 317:109. doi: 10.1007/s00403-024-03578-w, PMID: 39666160

[109] Napolitano M Esposito M Fargnoli MC Girolomoni G Romita P Nicoli E . Infections in patients with atopic dermatitis and the influence of treatment. Am J Clin Dermatol. (2025) 26:183–97. doi: 10.1007/s40257-025-00917-z, PMID: 39915363 PMC11850493

[110] Drucker AM Lam M Prieto-Merino D Malek R Ellis AG Yiu ZZN . Systemic immunomodulatory treatments for atopic dermatitis: living systematic review and network meta-analysis update. JAMA Dermatol. (2024) 160:936–44. doi: 10.1001/jamadermatol.2024.2192, PMID: 39018058 PMC11255974

[111] Partalidou S Patoulias D Deuteraiou K Avgerou P Kitas G Tzitiridou-Chatzopoulou M . Risk of major adverse cardiovascular events and venous thromboembolism with JAK inhibitors versus TNF inhibitors in rheumatoid arthritis patients: A systematic review and meta-analysis. Mediterr J Rheumatol. (2024) 35:10–9. doi: 10.31138/mjr.171023.rof, PMID: 38756933 PMC11094442

[112] Rajasimhan S Pamuk O Katz JD . Safety of janus kinase inhibitors in older patients: A focus on the thromboembolic risk. Drugs Aging. (2020) 37:551–8. doi: 10.1007/s40266-020-00775-w, PMID: 32514874 PMC7387323

[113] Tang Z Mu Z Wang X Zhao Y . Association between oral JAK-1 inhibitors and infection risks in atopic dermatitis: a retrospective analysis of the FAERS database. Front Med. (2025) 12:1694688. doi: 10.3389/fmed.2025.1694688, PMID: 41255603 PMC12620211

[114] Mikhaylov D Ungar B Renert-Yuval Y Guttman-Yassky E . Oral Janus kinase inhibitors for atopic dermatitis. Ann Allergy Asthma Immunol. (2023) 130:577–92. doi: 10.1016/j.anai.2023.01.020, PMID: 36736457

[115] Narla S Silverberg JI . Efficacy and risk stratification of janus kinase inhibitors in the treatment of moderate-to-severe atopic dermatitis. Dermatitis. (2024) 35:S24–38. doi: 10.1089/derm.2023.0058, PMID: 37527229

[116] Leung DY Guttman-Yassky E . Deciphering the complexities of atopic dermatitis: shifting paradigms in treatment approaches. J Allergy Clin Immunol. (2014) 134:769–79. doi: 10.1016/j.jaci.2014.08.008, PMID: 25282559 PMC4186710

[117] Sardana K Sharath S Meena SK Khurana A Dhir B . A real-world study of tofacitinib in Indian patients with refractory moderate-to-severe atopic dermatitis, its economic considerations and immunological rationale. Indian Dermatol Online J. (2025) 16:420–5. doi: 10.4103/idoj.idoj_665_24, PMID: 40395576 PMC12088487

[118] Guttman-Yassky E Silverberg JI Thaçi D Papp KA Ständer S Beck LA . Upadacitinib treatment withdrawal and retreatment in patients with moderate-to-severe atopic dermatitis: Results from a phase 2b, randomized, controlled trial. J Eur Acad Dermatol Venereol. (2023) 37:2558–68. doi: 10.1111/jdv.19391, PMID: 37528500

[119] Silverberg JI Thyssen JP Paller AS Papp K Igarashi A Feeney C . Assessment of efficacy and safety outcomes beyond week 16 in clinical trials of systemic agents used for the treatment of moderate to severe atopic dermatitis in combination with topical corticosteroids. Am J Clin Dermatol. (2023) 24:913–25. doi: 10.1007/s40257-023-00809-0, PMID: 37695504 PMC10570226

[120] Reich K DeLozier AM Nunes FP Thaçi D Torres T Papp KA . Efficacy and safety of baricitinib in combination with topical corticosteroids in patients with moderate-to-severe atopic dermatitis with inadequate response, intolerance or contraindication to ciclosporin: results from a randomized, placebo-controlled, phase III clinical trial (BREEZE-AD4). Br J Dermatol. (2023) 189:23–32. doi: 10.1093/bjd/ljad096, PMID: 35484697

[121] Blauvelt A de Bruin-Weller M Gooderham M Cather JC Weisman J Pariser D . Long-term management of moderate-to-severe atopic dermatitis with dupilumab and concomitant topical corticosteroids (LIBERTY AD CHRONOS): a 1-year, randomised, double-blinded, placebo-controlled, phase 3 trial. Lancet. (2017) 389:2287–303. doi: 10.1016/S0140-6736(17)31191-1, PMID: 28478972

[122] de Bruin-Weller M Thaçi D Smith CH Reich K Cork MJ Radin A . Dupilumab with concomitant topical corticosteroid treatment in adults with atopic dermatitis with an inadequate response or intolerance to ciclosporin A or when this treatment is medically inadvisable: a placebo-controlled, randomized phase III clinical trial (LIBERTY AD CAFÉ). Br J Dermatol. (2018) 178:1083–101. doi: 10.1111/bjd.16156, PMID: 29193016

[123] Chen Q Cui L Hu Y Chen Z Gao Y Shi Y . Short-term efficacy and safety of biologics and Janus kinase inhibitors for patients with atopic dermatitis: A systematic review and meta-analysis. Heliyon. (2023) 9:e22014. doi: 10.1016/j.heliyon.2023.e22014, PMID: 38034798 PMC10685203

[124] Sedeh FB Henning MAS Jemec GBE Ibler KS . Comparative efficacy and safety of monoclonal antibodies and janus kinase inhibitors in moderate-to-severe atopic dermatitis: A systematic review and meta-analysis. Acta Derm Venereol. (2022) 102:adv00764. doi: 10.2340/actadv.v102.2075, PMID: 35818735 PMC9574696

[125] Haag C Alexis A Aoki V Bissonnette R Blauvelt A Chovatiya R . A practical guide to using oral Janus kinase inhibitors for atopic dermatitis from the International Eczema Council. Br J Dermatol. (2024) 192:135–43. doi: 10.1093/bjd/ljae342, PMID: 39250758

[126] Kataoka Y . Thymus and activation-regulated chemokine (CCL17) as a clinical biomarker in atopic dermatitis: significance and limitations in the new treatment era. Front Allergy. (2024) 5:1473902. doi: 10.3389/falgy.2024.1473902, PMID: 39917426 PMC11799291

[127] Hawkins K David E Glickman JW Del Duca E Guttman-Yassky E Krueger JG . Atopic dermatitis stratification: current and future perspective on skin and blood transcriptomic and proteomic profiling. Expert Rev Clin Immunol. (2024) 20:1083–8. doi: 10.1080/1744666x.2024.2323964, PMID: 38436065

[128] Thijs JL Strickland I Bruijnzeel-Koomen C Nierkens S Giovannone B Csomor E . Moving toward endotypes in atopic dermatitis: Identification of patient clusters based on serum biomarker analysis. J Allergy Clin Immunol. (2017) 140:730–7. doi: 10.1016/j.jaci.2017.03.023, PMID: 28412391

[129] Bjerre RD Bandier J Skov L Engstrand L Johansen JD . The role of the skin microbiome in atopic dermatitis: a systematic review. Br J Dermatol. (2017) 177:1272–8. doi: 10.1111/bjd.15390, PMID: 28207943

[130] Natarelli N Gahoonia N Sivamani RK . Bacteriophages and the microbiome in dermatology: the role of the phageome and a potential therapeutic strategy. Int J Mol Sci. (2023) 24:2695–705. doi: 10.3390/ijms24032695, PMID: 36769020 PMC9916943

[131] Khadka VD Key FM Romo-González C Martínez-Gayosso A Campos-Cabrera BL Gerónimo-Gallegos A . The skin microbiome of patients with atopic dermatitis normalizes gradually during treatment. Front Cell Infect Microbiol. (2021) 11:720674. doi: 10.3389/fcimb.2021.720674, PMID: 34631601 PMC8498027

[132] Chu DK Koplin JJ Ahmed T Islam N Chang CL Lowe AJ . How to prevent atopic dermatitis (Eczema) in 2024: theory and evidence. J Allergy Clin Immunol Pract. (2024) 12:1695–704. doi: 10.1016/j.jaip.2024.04.048, PMID: 38703820

[133] Bang CH Park CJ Kim YS . The expanding therapeutic potential of deucravacitinib beyond psoriasis: A narrative review. J Clin Med. (2025) 14:1745–58. doi: 10.3390/jcm14051745, PMID: 40095888 PMC11900575

[134] Li Y Ashuo A Hao M Li Y Ye J Liu J . An extracellular humanized IFNAR immunocompetent mouse model for analyses of human interferon alpha and subtypes. Emerg Microbes Infect. (2024) 13:2287681. doi: 10.1080/22221751.2023.2287681, PMID: 37994664 PMC10810641

[135] Zhou J Liang G Liu L Feng S Zheng Z Wu Y . Single-cell RNA-seq reveals abnormal differentiation of keratinocytes and increased inflammatory differentiated keratinocytes in atopic dermatitis. J Eur Acad Dermatol Venereol. (2023) 37:2336–48. doi: 10.1111/jdv.19256, PMID: 37326015

[136] He H Suryawanshi H Morozov P Gay-Mimbrera J Del Duca E Kim HJ . Single-cell transcriptome analysis of human skin identifies novel fibroblast subpopulation and enrichment of immune subsets in atopic dermatitis. J Allergy Clin Immunol. (2020) 145:1615–28. doi: 10.1016/j.jaci.2020.01.042, PMID: 32035984

[137] Xia D Wang Y Xiao Y Li W . Applications of single-cell RNA sequencing in atopic dermatitis and psoriasis. Front Immunol. (2022) 13:1038744. doi: 10.3389/fimmu.2022.1038744, PMID: 36505405 PMC9732227

[138] Boca S Berce C Jurj A Petrushev B Pop L Gafencu GA . Ruxolitinib-conjugated gold nanoparticles for topical administration: An alternative for treating alopecia? Med Hypotheses. (2017) 109:42–5. doi: 10.1016/j.mehy.2017.09.023, PMID: 29150291

